# Modulation of Electronic and Optical Anisotropy Properties of ML-GaS by Vertical Electric Field

**DOI:** 10.1186/s11671-017-2181-y

**Published:** 2017-06-14

**Authors:** Fei Guo, Yaping Wu, Zhiming Wu, Congming Ke, Changjie Zhou, Ting Chen, Heng Li, Chunmiao Zhang, Mingming Fu, Junyong Kang

**Affiliations:** 1 Department of Physics, Fujian Key Laboratory of Semiconductor Materials and Applications, Collaborative Innovation Center for Optoelectronic Semiconductors and Efficient Devices, Xiamen University, Xiamen, 361005 People’s Republic of China; 2 Department of Physics, School of Science, Jimei University, Xiamen, 361021 People’s Republic of China

## Abstract

We investigate the electric-field-dependent optical properties and electronic behaviors of GaS monolayer by using the first-principles calculations. A reversal of the dipole transition from E//c to E⊥c anisotropy is found with a critical external electric field of about 5 V/nm. Decomposed projected band contributions exhibit asymmetric electronic structures in GaS interlayers under the external electric field, which explains the evolution of the absorption preference. Spatial distribution of the partial charge and charge density difference reveal that the strikingly reversed optical anisotropy in GaS ML is closely linked to the additional crystal field originated from the external electric field. These results pave the way for experimental research and provide a new perspective for the application of the monolayer GaS-based two-dimensional electronic and optoelectronic devices.

## Background

As a typical two-dimensional (2D) material, graphene has rather unique and exceptional properties [1], which enables its superior performance in transistors and as electrochemical electrodes [2]. Nevertheless, for use in nanoelectronic devices, the lack of intrinsic band gap [3] essentially restricts its application in the traditional emitting devices. Even though with surface functionalization and external electric or strain field, very small band gap can be achieved [4–7]. In this context, the search of other 2D materials that may offer new opportunities for specific properties and applications is both of fundamental interest and technological significance.

Recently, a stable class of 2D metal dichalcogenide (MD) materials, GaX (X = S, Se), has attracted much attention due to their exotic physical and chemical properties, with great promise for applications in fields such as solar energy conversion and optoelectronics [8–11]. Layer GaX is constructed by four-atom planes covalently bonded in the sequence of X-Ga-Ga-X with a D_3h_ symmetry. Advanced applications often require materials with tunable and reversible electronic properties which can be deliberately modulated by external control parameters. Strain engineering has been identified as one of the promising routes to tune the electronic behavior and the electron energy low-loss spectra of GaS monolayer (ML) and other 2D materials [12]. As an alternative, an applied electric field or light offers a novel way to modify the electronic properties over a wide range [13, 14]. For example, a strong electric field perpendicular to the plane of bilayer graphene can induce a significant band gap [15, 16], and the bandgap can also be modulated for BN with two or more layers [17]. However, the effects of the external electric field on the electronic structures of 2D GaS ML are still unclear. In addition, an intrinsic large negative crystal field which exists in GaS ML results in an optical anisotropy that the absorption coefficient for E⊥c is about 10^3^ cm^−1^, a factor of 30 smaller than for E//c [18]. For optic materials, light emission polarization is closely related to the near band-edge transitions, occurring between the bottom of the conduction band and the top of the valence band. By employing an external electric field, the band structure and thus the optical properties of GaS ML can be conveniently modulated to meet the multiple demands of device applications.

To address this issue, we perform a theoretical prediction on the modulation of optical and electronic anisotropy on GaS ML. Optical absorption spectra for both E⊥c and E//c directions are calculated under various external electric fields. Band structure and orbit contributions are analyzed to explain the dependence of the dipole transition on the external electric field. Spatial distribution of the partial charge and charge density difference are further simulated, which show the interlayer coupling and asymmetry electronic structure induced by the vertical external electric field, and reveal the physical mechanism for the modulation of the optical and electronic anisotropy of GaS ML. The present results are beneficial to supply the theoretical guidance on the tunable electronic and optoelectronic devices based on 2D GaS material.

## Methods

We perform the density functional theory (DFT) calculations with the Vienna Ab-initio Simulation Package (VASP) code [19], using the projector-augmented wave pseudopotential method [20]. Exchange and correlation effects are treated by Perdew–Burke–Ernzerhof (PBE) generalized gradient approximation (GGA) [21]. Heyd-Scuseria-Ernzerhof (HSE) hybrid functional is used to provide quantitative estimates of the band gap [22]. A slab model of the GaS consisting of four atom layers in the order S-Ga-Ga-S is employed, and a 15-Å vacuum layer along the z direction are adopted to eliminate the interactions between the slabs. The Brillouin zone is sampled according to Monkhorst–Pack method [23]. A 27 × 27 × 1 *k*-point mesh is used to relax the single-layer GaS, and a cutoff energy of 450 eV is taken for expanding the wave functions into a plane-wave basis. The convergence for energy is chosen as 10^-5^ eV between two steps and the maximum Hellmann-Feyman force acting on each atom is less than 0.01 eV/Å upon ionic relaxation. Gaussian smearing is used to address how the partial occupancies are set for each wave function, and the width of smearing is 0.1 eV. The imaginary part of the dielectric function due to direction interband transitions is obtained using the Fermi golden rule [24]. During the calculation, the spin-orbit coupling (SOC) splitting is neglected due to its tiny effects on electronic and optical properties.

## Results and Discussion

**Fig. 1:**
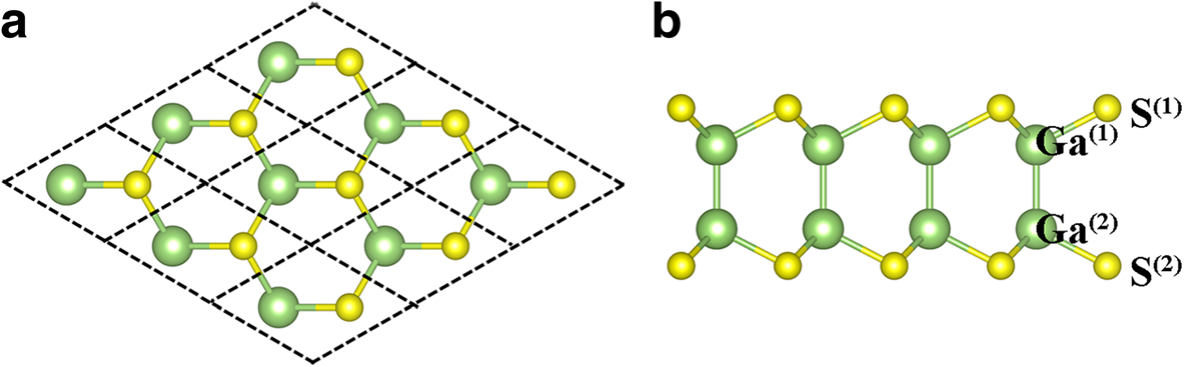
**a** Top and **b** side views of the atomic configuration of GaS ML. The *big green* and *small yellow spheres* represent Ga and S atoms, respectively, and the upper and lower interlayer atoms are labeled as Y^(1)^(Y = Ga, S) and Y^(2)^, respectively

Fully relaxed geometric configuration of GaS ML is shown in Fig. 1a, b. Monolayer thickness is calculated to be 4.66 Å, while the planar projection exhibits an ideal hexagonal honeycomb structure, similar to that of graphene. The lattice constant *a* is 3.64 Å, which is slightly larger than that of bulk material due to the lack of interlayer interaction [25]. The bond lengths of S–Ga and Ga–Ga are 2.37 and 2.48 Å, respectively, and the S–Ga–S angle between the nearest-neighbor S atoms are about 100.34°, which quite agree with the previous studies [12]. For convenience, the upper and lower interlayer atoms are labeled as Y^(1)^(Y = Ga, S) and Y^(2)^(Y = Ga, S), respectively.

**Fig. 2:**
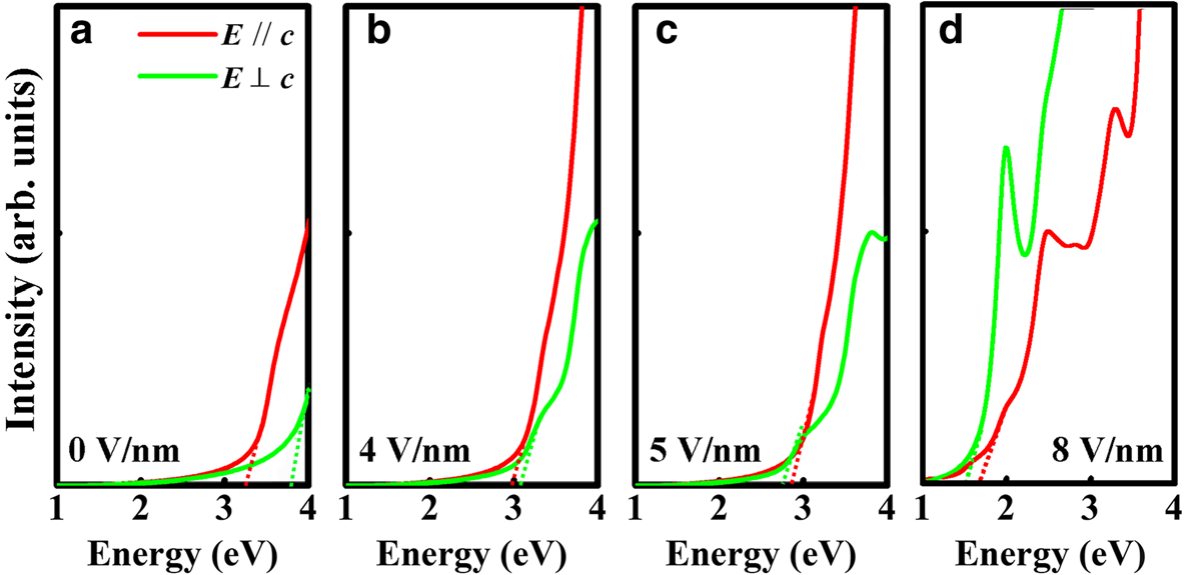
The calculated optical absorption spectra of the GaS ML **a** without an external electric field and **b**–**d** with an external electric field of 4, 5, and 8 V/nm, respectively. The absorption edge is labeled. *Red* and *green lines* represent TM and TE light, respectively

With the aim of modulating the optical properties of GaS ML, the optical absorption spectra with different external electric fields are calculated. The direction of applied electric field is along +z direction. As shown in Fig. 2, the absorption behaviors of extraordinary light (TM light; *E*//*c*) and ordinary light (TE light; *E*⊥*c*) are quite different, revealing the optical anisotropy in GaS ML. The absorption edge of TM and TE light are labeled by a red and green dash line, respectively. In the absence of an external electric field, the energy difference of absorption edge between TM light and TE light is approximately 0.55 eV (see Fig. 2a). As the external electric field is applied, both the absorption edges shift to the lower energy, and the energy difference of absorption edge decreases. A reversal of the dipole transition from *E*//*c* to *E*⊥*c* anisotropy occurs at a critical external electric field of about 5 V/nm. Note that the absorption edge of TE light is even lower than that of TM light as the electric field further increases to 8 V/nm. These results indicate that the optical anisotropy in GaS ML can be modulated by vertical external electric field.

**Fig. 3:**
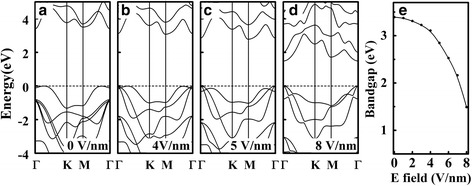
Band structure of GaS ML **a** without an external electric field and **b**–**d** with an external electric field of 4, 5, and 8 V/nm, respectively. The *dashed lines* indicate the Fermi levels, which are set to zero. **e** Variation of the energy gap with the external electric field for GaS ML

To gain an insight into the effects of external electric field on the optical anisotropy in GaS ML, the band structures without and with difference external electric fields are simulated. As shown in Fig. 3a, the conduction band minimum (CBM) of GaS ML is situated at Γ point, while the valence band maximum (VBM) locates at the position between Γ and *K* points, indicating an indirect bandgap. The DFT and the hybrid method calculated bandgap is 2.35 and 3.46 eV, respectively, which are in agreement with the previous results [12, 26]. Interestingly, in the presence of the external electric field E, as shown in Fig. 3b–d, the VBM switches to Γ point when E is beyond a critical value (about 5 V/nm), while the CBM still locates at the Γ point. This indicates an indirect to direct bandgap transition in GaS ML under the external electric field. In addition, as shown in Fig. 3e, the energy gap decreases monotonically with the increase of the external electric field. The bandgap modification arises from the well-known Stark effect, which has been observed in the previous studies on *h*-BN [27] and MoS_2_ [28]. When an external electric field is applied, there is a potential difference between the two interlayers (see Fig. 1b), which can be described as *U* = −*dE* ^*^ *e*, where *d* is the interlayer distance, and *E* ^*^ is the screened electric field. The external electric field elevates the potential of the lower interlayer and reduces that of the upper interlayer, resulting in a lifting of the VBM and a further decrease of the energy band gap. The stronger external electric field leads to the larger difference between the two interlayers, and thus the larger band splitting and smaller bandgap.

**Fig. 4:**
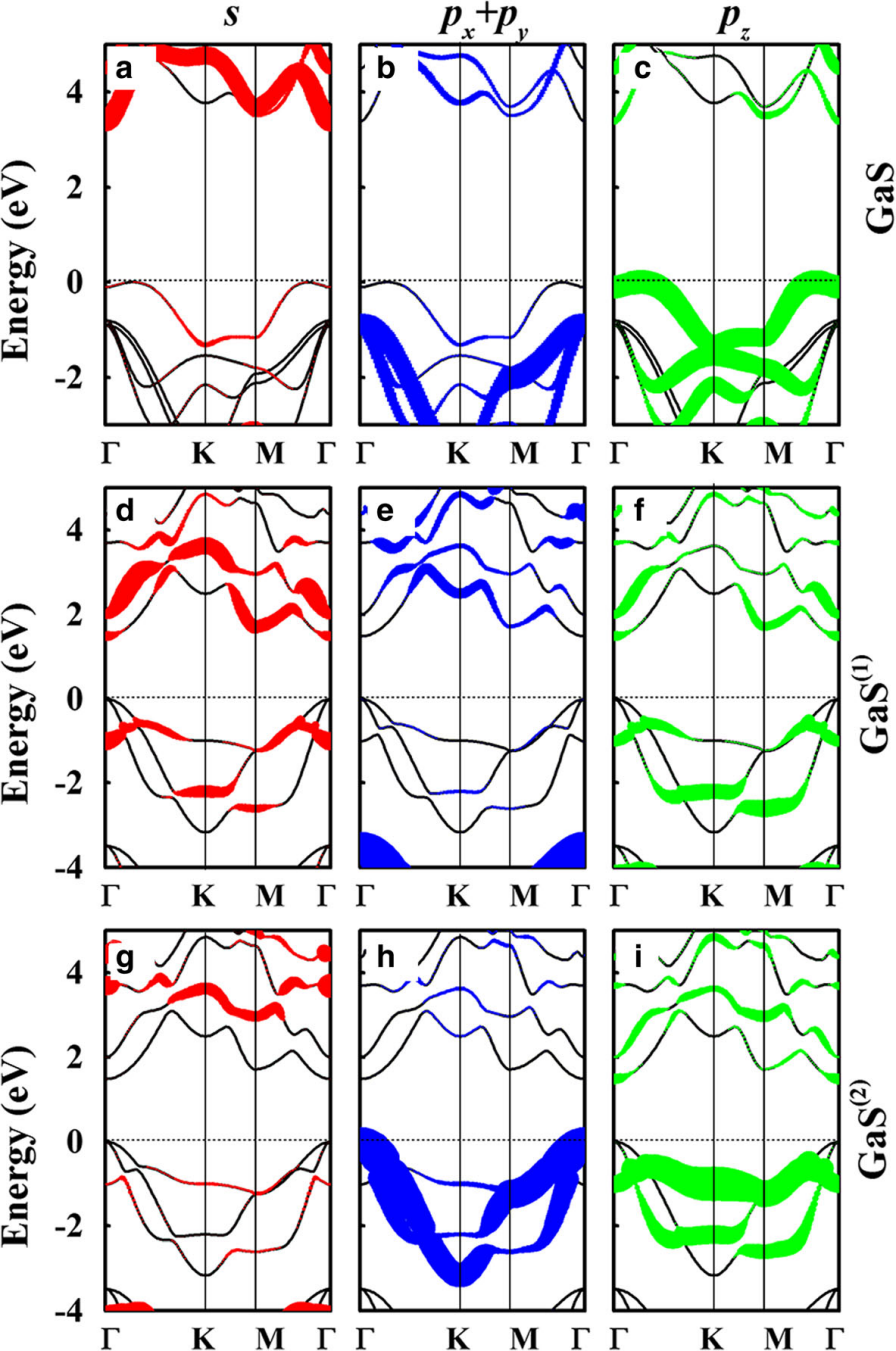
The decomposed projected band structure of the GaS ML. The top panel represent the s (**a**), p_x_ + p_y_ (**b**) , and p_z_ (**c**) orbits without an external electric field; the middle and last panels present the contributions of s (**d**, **g**), *p*_x_ + *p*_y_ (**e**, **h**), and *p*_z_ (**f**, **i**) orbits from the upper and lower interlayer of GaS with an external electric field of 8 V/nm, respectively

To reveal the evolution mechanism of the optical anisotropy of GaS ML, the decomposed projected band structures with and without an electric field are further calculated, as shown in Fig. 4. For the original GaS ML without the electric field, the CBM and VBM are mainly contributed by the hybridized *s* and *p*_*z*_ states of Ga atoms and the *p*_*z*_ states of S atoms, respectively, while the following four valence bands below the VBM are mainly composed of the in-plane *p*_*x*_ + *p*_*y*_ stats of S atoms. When an external electric field of 8 V/nm is applied, the upper and lower Ga-S layers exhibit asymmetrical contribution to the band structure. The CBM is mainly occupied by both the *s* and *p*_*z*_ orbital components of the upper Ga^(1)^S^(1)^ layer but only the *p*_*z*_ states of the lower Ga^(2)^S^(2)^ layer. Compared with that of the interlayer coupling states in the conduction band, the in-plane states in the valence band are even more sensitive to the vertical external electric field. It is found that the *p*_x_ + *p*_y_ states of the upper Ga^(1)^S^(1)^ and lower Ga^(2)^S^(2)^ layers possess separate lower and higher energies, respectively, and the energy difference at Γ point is about 3.05 eV. This indicates that the external electric field induces asymmetric electronic structures in GaS interlayers. The uplifted *p*_x_ + *p*_y_ states of the lower Ga^(1)^S^(1)^ layer surpass the *p*_*z*_ states of the S atoms and become the uppermost valence band, leading to a replacement of the VBM, from the original point between Γ and K to the Γ point. This change of the VBM results in the evolution of the dipole transition from *E*//*c* to *E*⊥*c* preference, which explains the above prediction that the absorption of *E*⊥*c* is gradually increased with the vertical external electric field and exceed that of *E*//*c* at a critical external electric field of about 5 V/nm.

**Fig. 5:**
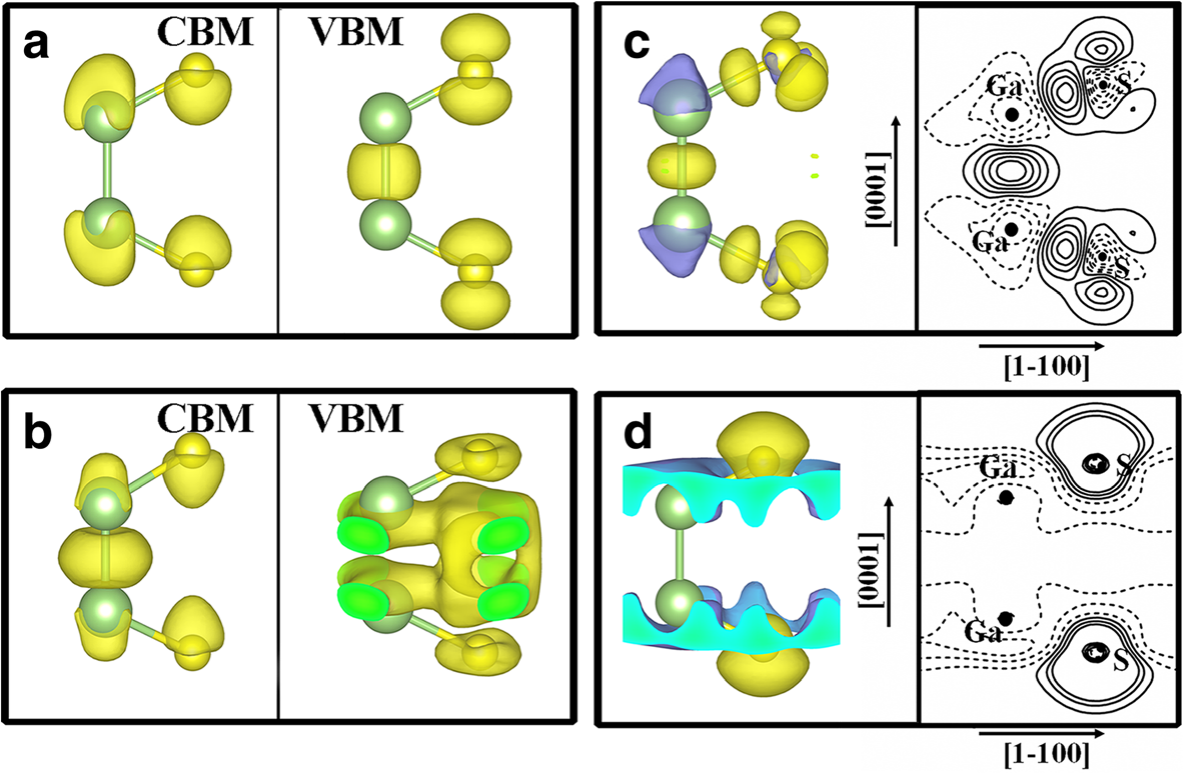
Partial density of states of the CBM and VBM of GaS ML without (**a**) and with (**b**) an external electric field of 8 V/nm, respectively. Spatial charge density difference and the vertical section along (1-100) plane of GaS ML without (**c**) and with (**d**) an external electric field of 8 V/nm, respectively. The positive and negative density (contours) are, respectively, shown with *yellow* (*solid lines*) and *blue* (*dashed lines*) colors, and the contour interval is 0.005 eÅ^−3^

Spatial distribution of the partial charge at CBM and VBM of GaS ML is further calculated without and with an external electric field of 8 V/nm, as shown in Fig. 5a, b, respectively. The CBM of both cases have a *s*-type state character that is tightly localized around the S atoms in a spherical shape. While at lower electric fields (0~5 V/nm), the VBM is only from a *p*_*z*_ state distributing as a dumbbell shape parallel to the z direction. As the external electric field is increased to the critical value and larger, the VBM is derived from the mixture of *p*_*x*_ and *p*_*y*_ components, exhibiting another dumbbell shape perpendicular to the z direction. Based on the VB ordering, parity selection rules come into play. Interband transitions under *xy* polarization are only allowed for the states with the same parity, whereas those under z-polarization are uniquely for states with opposite parities. Therefore, with an external electric field from 0 to 5 V/nm, the lowest transition CBM-VBM in GaS are only available for the TM-polarized light (*E*//*c*), while as the external electric field is larger than 5 V/nm, the lowest transition CBM-VBM tunes to be available for the TE-polarized light (*E*⊥*c*) only. This phenomenon pronounces a modulation of the electronic and optical anisotropy under a vertical electric field. The origin of the opposite optical anisotropy can be traced back to the additional crystal field induced by the electric field, as evidenced by the charge density difference plotted in Fig. 5c, d. Without the external electric field, electrons are observed to accumulate in the Ga–S and Ga–Ga binding regions, forming ionic bonds and covalent bonds, respectively. When applying an external electric field, more and more electrons tend to accumulate around the S atoms, while less and less electrons distribute between the upper and lower Ga atoms. This means that the external electric field reduces the interaction between the upper and lower interlayers in GaS and enhances the interaction between S and Ga atoms within each interlayer; as a result, an electron transport channel is created above the electric field of 5 V/nm, such as 8 V/nm in Fig. 5d. The above analysis indicates that the strikingly reversed optical anisotropy in GaS ML is closely linked to the additional asymmetric crystal field originated from the external electric field applied.

## Conclusions

In summary, based on the first-principles DFT simulations, we investigate the electric-field-dependent optical properties and electronic behaviors of GaS ML. Optical absorption spectra for both *E*⊥*c* and *E*//*c* directions are calculated under various external electric fields. A reversal of the dipole transition from *E*//*c* to *E*⊥*c* anisotropy is found with a critical external electric field of about 5 V/nm. The band structure calculations indicate a reduction of the band gap and a transition from indirect to direct bandgap in GaS ML with an increasing external vertical electric field. Decomposed projected band contributions exhibit the asymmetric electronic structures in GaS interlayers under the external electric field, which explains the evolution of the absorption preference. Spatial distribution of the partial charge and charge density difference suggest that the strikingly reversed optical anisotropy in GaS ML is closely linked to the additional crystal field which originated from the external electric field. These results not only reveal the modulation of the electronic structures and optical properties of GaS ML by the external electric field but also provide some references to its future application in 2D electronic and optoelectronic devices.
